# Evaluation of educational resource utilization efficiency, regional technological heterogeneity, and total factor productivity change in 35 European countries

**DOI:** 10.1371/journal.pone.0295979

**Published:** 2024-01-19

**Authors:** Huayue Liao, Gang Hao, Rizwana Yasmeen, Wasi Ul Hassan Shah

**Affiliations:** 1 School of Management, Zhejiang Shuren University, Hangzhou, China; 2 Department of Management Sciences, City University of Hong Kong, Kowloon, China; 3 School of Economics and Management, Panzhihua University, Panzhihua, Sichuan, China; 4 Department of Economics, University of Religions and Denominations, Qom, Iran; National Kaohsiung University of Science and Technology / Industrial University of Ho Chi Minh, TAIWAN

## Abstract

Educational resource utilization efficiency (ERUE) and productivity growth are considered current global challenges that the modern world faces. This study evaluates the educational resource utilization efficiency, dynamic productivity change, and regional discrepancies in technologies involved in educational resource utilization in 35 European countries and four regions. DEA Super SBM, Meta frontier analysis, and Malmquist productivity index are employed to gauge the ERUE, technology gap ratio (TGR), and total factor education resources productivity change. A set of inputs and outputs is used from 35 European countries for the study period of 1998–2021. Results revealed that the average ERUE in European countries is 0.6312, Which indicates a 36.88% improvement potential in educational resource utilization. Southern Europe continuously exhibits superior average ERUE scores (0.6871) compared to other regions, indicating a higher efficiency in using educational resources. Luxembourg (1.0813), Czechia (0.9356), and Slovenia (0.8984) are found to be the top three performers in terms of ERUE level. The technology gap ratio value is highest in Southern Europe. It demonstrates that southern European countries used the most advanced technology in education resource utilization. The average Malmquist Index (MI) in European countries is 1.0349. It Indicates a 3.49% growth in educational resource utilization. Technology is the primary determinant of productivity growth, as Technological change is higher than efficiency change. Southern European countries showed the highest MI of 1.0542. Italy, Lithuania, and Serbia were found to have higher average MI scores over the study period (1998–2021). Finally, the Kruskal Wallis test proved that ERUE and TGR in 4 different regions of Europe are heterogeneous. In contrast, the MI in European regions isn’t found to be significantly different.

## 1. Introduction

Education provides the skills and critical thinking needed in ever-changing societies. Education boosts productivity, creativity, and competitiveness, improving employment access, inequality, and foreign investment [1, 2]. Education empowers marginalized communities and promotes inclusivity, civic involvement, and empathy, uniting various cultures [3]. Education helps people participate and adapt to global issues like healthcare, sustainability, and technology. Education shapes society and promotes economic prosperity, peace, and a better future [4]. Quality education is crucial to nation-building. It gives citizens the knowledge, skills, and critical thinking to boost economic growth, innovation, and social change. A well-educated population creates a skilled workforce, attracts investments, and encourages entrepreneurship, which enhances industries and worldwide competitiveness [5]. Quality education boosts community engagement, informs decision-making, and improves democratic institutions, promoting stable governance and inclusive growth. Quality education empowers individuals to contribute meaningfully to their country’s growth and ensures a brighter future for future generations [6].

Due to abundant resources, advanced technology, and qualified educators, developed countries’ educational institutions offer innovative curricula and diversified opportunities. Educational gaps arise from low funding, overcrowded schools, and scarce resources in underdeveloped countries [7, 8]. Despite these challenges, many developing countries are improving their systems through inclusive legislation, teacher training, and curricular revisions, showing resilience and a dedication to learning. Quality education is essential for social and economic success; bridging different systems is critical for global prosperity [9]. The efficient utilization of educational resources varies across nations, reflecting their priorities and strategies. Developed countries frequently allocate substantial funds to education to provide modern facilities, modernized technology, and well-trained educators [10–12]. These nations emphasize research, innovation, and interdisciplinary learning to maximize resource impact. In contrast, developing nations confront resource limitations but strive for efficiency through targeted investments, community engagement, and adaptable teaching methods [13]. Numerous countries partner with international organizations or digital platforms to broaden educational access. Despite divergent approaches, nations recognize that optimizing resource utilization is essential for empowering learners, driving socio-economic growth, and fostering a globally competitive workforce [14].

The development of a high-quality education system has underpinned Europe’s progress. Numerous European nations have well-established educational systems with rigorous standards, highly trained educators, and a comprehensive curriculum [15, 16]. These Countries prioritize inclusive and accessible education, frequently providing citizens and international students with free or low-cost higher education options. The European Higher Education Area (EHEA) encourages harmonizing educational systems, student mobility, and international collaboration [17]. In addition, innovative teaching methods, advanced research facilities, and digital integration contribute to Europe’s academic reputation. Consequently, European nations continue to shape a dynamic educational landscape that fosters critical thinking and cultural awareness and prepares students to excel in a constantly evolving global context [18, 19]. However, efficiency in allocating educational resources varies across European nations, reflecting diverse approaches and priorities. Nordic nations such as Finland and Sweden are renowned for their equitable distribution of resources, which places a premium on teacher training, individualized instruction, and student welfare [20]. Germany places a high priority on vocational education, combining classroom instruction with training in practical skills. In contrast, Southern European countries struggle with resource allocation due to economic constraints, resulting in disparities in infrastructure and class sizes [21]. Increasingly, Eastern European nations are investing in education, leveraging technology and international partnerships to improve resource efficiency. The commitment to quality education, tailored to each country’s context, to cultivate a skilled workforce and societal development is a unifying factor among diverse approaches [22, 23].

EU member states are working to improve educational resource utilization. The EU has launched numerous measures to optimize resource allocation and use in its different educational systems, recognizing education’s vital role in economic growth, social cohesion, and individual empowerment [24]. These programs promote member-state information sharing and best practices. Innovative teaching methods, digital technologies, and pedagogical tactics are shared on EU platforms. Educators can improve resource efficiency and student performance by collaborating internationally. The EU also promotes digital technology in education [25]. Member states have received capital and policy support for digital curriculum delivery, evaluation, and administration tools. This streamlines resource allocation and improves education, especially in remote or impoverished areas [26]. The European Union has actively advocated for Open Educational Resources (OER) and materials with open access. Through the transparent sharing of educational content among member states, there is an opportunity to minimize redundancy, decrease textbook costs, and enhance the availability of learning materials. This initiative aligns with the EU’s objectives of democratizing and fostering inclusivity in education [27]. The EU promotes education resource efficiency in its member states through collaborative research, policy coordination, and targeted funding. The EU uses innovation, technology, and common expertise to improve education and prepare its inhabitants for the future. Educational resources, geographical diversity, and technology variety across Europe create significant opportunities and difficulties. Educational resources vary widely by geography, typically reflecting economic and historical differences [28, 29].

Schools in more developed areas have superior funding, facilities, and resources. It may improve education, educational materials, and technology access. Poorer or distant areas may lack resources, facilities, and educational technology [30, 31]. European regional and cultural variety affects educational agendas and curriculum needs. Countries may stress different subjects or talents based on their economies, morals, and histories. Diversity broadens pupils’ perspectives and emphasizes the need for specialized resource allocation and curriculum improvement [32]. Technology heterogeneity is noteworthy. Some European countries have embraced digital innovation and integrated technology extensively into their education systems. Still, others struggle with unequal access to technology, varying levels of digital literacy among educators and students, and different technology integration policy frameworks [33]. National and international activities address these inequities. The European Union and the Council of Europe have funded projects to reduce educational inequality, share best practices, and promote digital literacy between member states. Cross-border partnerships and OER sharing can help close gaps and distribute educational advantages more evenly [34]. European countries take numerous steps to enhance educational resources utilization efficiency (ERUE), and productivity growth and minimize the regional discrepancy in their technological usage in education sectors [35].

However, the level of success in the mission of educational resource utilization efficiency, productivity change, and regional technological heterogeneity gaps in Europe is undiscovered and worth comprehensive investigation. To this end, in the first stage, this study applied the DEA Super-SBM model to estimate educational resources utilization efficiency (ERUE) for a well-stretched period of 1998–2021. It explores the level of accomplishment in the ERUE enhancement program over the study period in different European countries and regions. The super SBM model also distinguishes among the efficient countries. The study employed the Meta-frontier analysis in the second stage to gauge the technological gap ratio in four regions (Northern, Eastern, Western, and Southern Europe). Thirdly, the study used the Malmquist productivity index to gauge the total factor of educational productivity in different European countries. It evaluates the main determinant of productivity change (efficiency or technological change) over the study period. Finally, the Kruskal-Wallis test is employed to gauge the statistically significant difference among 4 European regions for mean RUE, MI, and TGR. The rest of the study is organized as follows. Section 2 illustrates the comprehensive, relevant literature review. Section 3 and 4 describes the methodology used in the study and Variables selection and data collection. The results and discussion are discussed in section 5. Section 6 illustrates the conclusion and policy implications.

## 2. Literature review

Numerous research studies evaluate the efficiency and productivity of educational resources in different countries and regions. Guarini et al. [36] examined how private and public resources impact educational achievement in Italy, utilizing Sen’s capability approach. They analyzed a 1993–2012 longitudinal dataset derived from the Italian Statistical Institute’s social survey and employed a panel stochastic frontier model to address endogeneity. Their study revealed the importance of private wealth, the synergy between it and public education spending, and the positive effects of social capital and government quality on efficient educational resource allocation. Similarly, Higgins et al. [37] summarize recent research from Heriot-Watt University and the UK Center for Education in the Built Environment, focusing on the challenges of balancing effective teaching and resource efficiency in formative assessment. This study emphasizes the benefits of formative assessment, where students receive feedback for improvement without grading. The research demonstrates the positive impact of formative assessment on higher education learning.

Using Data Envelopment Analysis (DEA) and two-way fixed-effect panel data analysis, the study found that East and Southeast Asia have the most efficient educational systems among the 53 countries studied. The study underscores the varying impact of education on competitiveness based on a country’s development stage and location. It suggests that governments should align growth priorities with their developmental stage and regional context while considering competitiveness [38]. Veremchuk et al. [39] assessed ten educational libraries and found that users have varied objectives, with students seeking textbooks and interaction, while scholars prioritize reference, research, and electronic resources. Bhutoria and Aljabri [40] analyze school resource and personnel management efficiency in OECD and MENA countries using TIMSS 2019 data from 5,164 schools across 26 nations. They find technical inefficiencies in schools in both regions, highlighting that educational resources alone do not guarantee improved learning outcomes. The study underscores the importance of effective educational management systems, student discipline, and personnel management in enhancing school-level efficiency. It recommends prioritizing school-level administration, discipline, and stakeholder engagement to improve technical efficiency rather than solely focusing on increasing resources. Nojavan et al. [41] assess technical inefficiency in school resource and personnel management in OECD and MENA countries. They find inefficiencies in schools and stress the need for effective educational management and discipline, emphasizing school-level administration and stakeholder engagement over resource quantity.

In another, the relationship between ICT adoption and school efficiency is examined using 2018 PISA data from 5,400 schools. The analysis reveals low ICT efficiency in schools, mainly attributed to their ability to convert ICT-mediated instructional time into effective learning experiences, highlighting challenges in transitioning to digital learning environments [42]. Delahoz-Dominguez et al. [43] use three DEA methods to assess academic efficiency in Colombian engineering schools. They find that 14.3%, 29.8%, and 88.7% of programs are efficient. This study uniquely integrates high school and college standardized test results as input variables, emphasizing quality and efficiency. It demonstrates that more efficient colleges can optimize human resources and highlights the positive impact of high-quality credentials on university productivity. The research introduces an analytical methodology for evaluating degree programs, aiding informed decision-making in education. Munoz and Queupil [44] evaluate the effectiveness of Chilean secondary education institutions following educational reforms. They use Data Envelopment Analysis (DEA) to create an efficiency index, comparing schools based on student performance and socio-economic variables. Findings indicate private schools are more efficient, and publicly sponsored schools outperform public ones when considering socio-economic factors. This study informs Chilean educational policymaking, focusing on resource allocation for enhanced quality and equity in secondary education. It emphasizes the importance of education in social mobility and economic growth, offering a unique and comprehensive analysis with practical implications for decision-makers.

This study introduces a data envelopment analysis (DEA) method to assess university teaching and research efficiency. It identifies 16 key performance measures and uses joint DEA maximization for evaluation. A hypothetical case demonstrates the model’s effectiveness, and the study explores its potential for university performance improvement and resource optimization, benefiting academics and administrators [45]. Xu and Liu [46] introduce a novel method for assessing the efficiency of education and technology within national science and education departments. Using Data Envelopment Analysis (DEA) and panel data from 53 countries, the study reveals efficiency progress in East Asian countries and some developing nations. It also explores the impact of educational and technological efficiencies on national competitiveness, balanced development, energy efficiency, export, and employment. Findings indicate that technological efficiency promotes balanced development, while educational efficiency hinders it. The study further highlights the evolving contributions of educational and technological efficiency to development, influenced by economic development and policies from 2000 to 2014. This study contributes valuable insights to inform policymaking in education and technology, building upon relevant findings in the field [47–55].

## 3. Methodology

Data Envelopment Analysis (DEA) is a commonly used linear programming technique employed to assess the relative efficiency of decision-making units (DMUs) within various industries [56, 57]. The selection of Data Envelopment Analysis (DEA) over Stochastic Frontier Analysis (SFA) is due to DEA’s non-parametric nature, flexibility with diverse inputs/outputs, ability for relative efficiency comparison, and ease of interpretation. The foundational constant return to scale model (CSR) was introduced by Charnes et al. [58], and later, Banker et al. [59] modified it to the variable return to scale (VSR) model. Building upon DEA methodology, Tone [60] further contributed to the field by proposing the Slack-based Measure (SBM) model.

### 3.1 DEA Super-SBM model

The CCR-based radial DEA model falls short of comprehensively addressing the impact of idleness on productivity. In response to this limitation, Tone [60] introduced the SBM (Slacks-Based Measure) and super-efficiency SBM models as alternative methodologies. SBM represents a non-radial approach to assess efficiency in scenarios where input and output do not vary proportionally [61]. By amalgamating the concepts of super-efficiency and SBM, the super-efficiency SBM model emerges as a robust modeling framework.

The core premise of the super-efficiency evaluation method involves isolating the efficient evaluation unit from the set and appraising it independently. Consequently, the original assessments of non-efficient values remain unchanged, while evaluations of efficient values may exceed 1. The SBM model offers an expedient solution for addressing input excess and output deficiency promptly. In the SBM model, the data unit remains constant, enabling the systematic adjustment of input and output slack variables to compensate for deficiencies in an equitable manner. A notable advantage of the SBM model over its counterparts lies in its precision when evaluating the efficiency of less efficient DMUs (Decision-Making Units).

In the context of this formulation, we have a set of decision-making units (DMUs) denoted by η, each comprising input and expected output vectors. Specifically, we have three vectors $x\in R^{M},y^{g}\in R^{S1},y^{b}\in R^{S1}$, representing the expected output of **S**_**1**_ given **m** units of input. It’s important to note that all these vectors are assumed to be positive **X**>**0, Y**^**g**^**>0, Y**^**b**^**>0**.

The production possibility set is defined as follows:

$$=[x_{1},x_{2},\ldots ,x_{N}]\in R^{N\times M},$$

$$Y^{g}=[y^{g1},y^{g2},\ldots ,y^{gN}]\in R^{S1\times N}.$$

Now, let’s consider the set P, which captures the relationship between these vectors and their optimal counterparts:

$$P=\{(x,y^{g},y^{b})|,x\geq X\eta |,y^{g}\leq Y\eta |,y^{b}\geq Y\eta |,\eta \geq 0\}$$ (1)

In Eq (1), it’s implied that the actual expected output (**x**_**0**_**y**^**g**^_**0**_**,y**^**b**^_**0**_) for a given DMU (x) may fall short of the optimal expected output achievable on the frontier. Tone’s SBM (Slacks-Based Measure) model takes this into account by considering the slack or the difference between the actual and optimal outputs when assessing a DMU.

The SBM model incorporates the production possibility set to evaluate how efficiently a DMU operates relative to its full potential, considering both inputs and outputs. This allows for a more nuanced assessment, acknowledging that DMUs might not always achieve their optimal levels of production or output efficiency.


$$\gamma =min(\frac{1-\frac{1}{M}\sum_{i=1}^{M}\frac{S_{i}^{-}}{x_{io}}}{1+\frac{1}{S1+S2}(\sum_{r=1}^{S1}\frac{S_{r}^{g}}{y_{r0}^{g}}+\sum_{r=1}^{S2}\frac{S_{r}^{b}}{y_{r0}^{b}})})$$ (2)

$$s.t.\left\{ \begin{array}{c} x_{0}=X\eta +S^{-}\\ y_{0}^{g}=Y^{g}\eta -S^{g}\\ y_{0}^{b}=Y^{b}\eta +S^{b}\\ S^{-}\geq 0,S^{g}\geq 0,S^{b}\geq 0,\eta \geq 0 \end{array}\right.$$

In Eq (2), the symbol γ represents the efficiency of the DMU, and its value falls within the range of 0 to 1. The variables (**S**^−^**S**^−^,**S**^**g**^,**S**^**b**^) correspond to input, output, and slack, respectively. A DMU is considered to be operating at the frontier of production only when its technical efficiency ***γ***) is equal to 1, and l three slack variables **S**^−^,**S**^**g**^,**S**^**b**^ are equal to 0. When ***γ*** is less than 1, it indicates that the DMU is operating inefficiently. To transform the nonlinear Eq (2) into a linear model, you can employ the Charnes-Cooper transformation. This transformation is a common approach in Data Envelopment Analysis (DEA) to linearize the model and facilitate efficiency evaluations. It allows for a more straightforward analysis of the relative efficiency of DMUs by introducing additional variables and constraints. These linearized models can be solved using linear programming techniques.


$$\kappa =m\;(T-\frac{1}{M}\sum_{i=1}^{M}\frac{S_{i}^{-}}{x_{io}})$$ (3)

$$\left\{ \begin{array}{c} 1=T+\frac{1}{S1+S2}(\sum_{r=1}^{S1}\frac{S_{r}^{g}}{y_{ro}^{g}}+\sum_{r=1}^{S2}\frac{S_{r}^{b}}{y_{ro}^{b}})\\ x0T=X\beta +S^{-}\\ y_{0}^{g}T=Y^{g}\beta -S^{g}\\ y_{0}^{b}T=Y^{b}\beta +S^{b}\\ S^{-}\geq 0,S^{g}\geq 0,S^{b}\geq 0,\beta \geq 0,T\geq 0 \end{array}\right.$$

Indeed, in some cases, certain decision-making units (DMUs) can be effective at assessing the technological efficiency of alternatives. To address these situations and provide a fairer approach to efficiency measurement, the Super SBM model (Super-Efficiency Slacks-Based Measure model) was developed by building upon existing research. This model extends the previous work in order to offer a more comprehensive and equitable method for evaluating efficiency.


$$\gamma^{*}=m\;\left[\frac{\frac{1}{M}\sum_{i=1}^{M}\frac{\bar{x}i}{\frac{x}{0}0}}{\frac{1}{S1+S2}(\sum_{r=1}^{S1}\frac{{\bar{y}}_{r}^{s}}{y_{ro}^{g}}+\sum_{r=1}^{S2}\frac{{\vec{y}}_{r}^{b}}{y_{ro}^{b}})}\right]$$ (4)

$$\left\{ \begin{array}{c} \bar{x}\geq \sum_{j=1,\neq 0}^{N}\eta_{j}x_{j}\\ {\bar{y}}^{g}\leq \sum_{j=1,\neq 0}^{N}\eta_{j}y^{g}{}_{j}\\ {\vec{y}}^{b}\geq \sum_{j=1,\neq 0}^{N}\eta_{j}y^{b}{}_{j}\\ \bar{x}\geq x0,{\bar{y}}^{g}\leq y_{0}^{g},\vec{y^{b}}\geq y_{0}^{b},{\bar{y}}^{g}\geq 0,\eta \geq 0, \end{array}\right.$$

The super-efficiency of a decision-making unit (DMU) is represented by γ* in Formula (4), and it is possible for γ* to exceed 1. This signifies that the DMU is not only efficient but is operating at a level of efficiency that surpasses the efficiency of the most efficient DMUs in the dataset. In other words, a γ* value greater than 1 indicates that the DMU is exceptionally efficient compared to its peers and represents a benchmark for others to strive towards.

### 3.2 DEA-meta frontier model

The Meta-frontier Model provides greater precision in estimating DMU (Decision Making Unit) efficiency when comparing various groups. For an objective evaluation of DMUs within the same group, where all entities have access to the same technology, it is therefore recommended to conduct comparisons within that group. The Technological Gap Ratio (TGR) is a valuable tool for measuring the technological advancement disparity between distinct groups. This TGR can be presented to a specific audience to illustrate the disparities between the groups’ technological development [62].


$$TGR=\frac{MERUE}{GERUEi}$$ (5)

In this context, *GERUEi* represents the educational resources utilization efficiency (ERUE) of Decision-Making Units (DMUs) belonging to a particular group. Conversely, MERUE represents the Meta-ERUE, which encompasses DMUs from the entire population, spanning all groups. The Technology Gap Ratio (TGR) serves as a metric for quantifying the disparity between these two categories of DMUs. It assesses how the technology level of the meta-frontier (comprising all DMUs) compares to the technology level of a specific group’s frontier. When the TGR has a value of 1, it signifies that there is no technological gap or difference between the group’s performance and the meta-frontier. In other words, the group’s technology is on par with the overall average technology level [63].

### 3.3 DEA-Malmquist productivity index

The Malmquist productivity indices provide Decision-Making Units (DMUs) with a means to monitor the progress of efficiency enhancements across time. Nevertheless, the reliability of these indices is contingent upon the assumption that the production function faithfully reflects the prevailing technological condition. DEA models are employed to pinpoint the location of this efficiency boundary, offering insights into how efficiently resources are being utilized and highlighting areas for potential improvement. The characteristics of a particular DMU (*DMU*_0_) are defined by the output variation between two consecutive periods, "t" and "t+1" [64].


$$M_{0}=\frac{D_{0}^{t+1}(x_{0}^{t+1},y_{0}^{t+1})}{D_{0}^{t}(x_{0}^{t}y_{0}^{t})}{\left[\frac{D_{0}^{t}(x_{0}^{t+1},y_{0}^{t+1})}{D_{0}^{t}(x_{0}^{t},y_{0}^{t})}\frac{D_{0}^{t+1}(x_{0}^{t+1},y_{0}^{t+1})}{D_{0}^{t+1}(x_{0}^{t}y_{0}^{t})}\right]}^{1/2}$$ (6)

- Where: $D_{0}^{t}(x_{0}^{t},y_{0}^{t})$ indicate the TE estimation of *DMU*_0_ for period t,
- $D_{0}^{t+1}(x_{0}^{t+1},y_{0}^{t+1})$ indicate the TE measurement of *DMU*_0_ for period t+1.
- $D_{0}^{t}(x_{0}^{t+1},y_{0}^{t+1})$ shows the change in TE from t to t+1.
- The TE of a specific DMU (*DMU*_0_) at time t+1, denoted as $D_{0}^{t+1}(x_{0}^{t},y_{0}^{t})$, is determined by using its data from period t and comparing it with the data from period t+1.

### 3.4 Kruskal–Wallis test

Kruskal-Wallis is a non-parametric statistical test used to compare three or more independent samples to determine whether they originate from the same population or have statistically significant differences. It is frequently used when the assumptions of normality and homogeneity of variances required by conventional analysis of variance (ANOVA) tests are not satisfied. The test, named after its creators William Kruskal and W. Allen Wallis, ranks the values from all the samples and then calculates a test statistic based on the ranks [65]. This test statistic follows a chi-squared distribution, and its significance assists researchers in determining whether differences between sample medians are likely due to chance or represent meaningful distinctions. If the Kruskal-Wallis test reveals a statistically significant difference between groups. This study employed the Kruskal-Wallis test to gauge the Statistical Significant difference among average ERUE, MI, and TGR in four regions of Europe.

H_01_: The average ERUE scores are the same across four different European regions.

H_02_: The average MI scores are the same across four different European regions.

H_03_: The average TGR scores are the same across four different European regions.

## 4. Variables selection and data collection

Inputs-outputs selection in DEA estimation has great importance for the credibility of results [66–68]. Table 1 clearly shows the factors carefully chosen to determine how well educational resources are being used. Evaluating the efficiency of educational resources is important for knowing how well different inputs lead to the desired educational results. The following variables are being selected based on previous studies that used DEA to gauge the efficiency of educational resources [69, 70]. One of the input factors is "Government Expenditure on Education, Total (% of Government Expenditure)," which shows how much of a country’s total government spending goes to education. It shows how committed the government is financially. The "Number of Teachers in Primary Education," "Number of Teachers in Secondary Education," and "Number of Teachers in Upper Secondary Education" numbers also show how many people are working as teachers at different levels.

**Table 1:** Variables employed to estimate the educational resources utilization efficiency.

Inputs	Outputs
Government expenditure on education, total (% of government expenditure)	Literacy rate, youth (ages 15–24)
No. of Teachers in Primary Education	Educational attainment, at least completed upper secondary, population 25+, total (%) (cumulative)
No. of Teachers in Secondary Education	
No. of Teachers in upper secondary education	

On the other hand, the output factors include "Literacy Rate, Youth (Ages 15–24)," which shows how well young people can read and write, and "Educational Attainment, At Least Completed Upper Secondary, Population 25+, Total (%)," which shows how well adults did in school. By looking at these factors together, stakeholders can learn much about how well educational resources are used to reach educational goals. It helps them make smart choices about how to improve the system.

For the purpose of figuring out how well educational resources are being used, Table 2 divides 35 European countries under evaluation into four regions: Northern Europe, Eastern Europe, Western Europe, and Southern Europe. The data for these 35 countries was collected from world development indicators (WDI) for 1998–2021. Iceland, Bosnia and Herzegovina, Liechtenstein, Malta, Montenegro, North Macedonia, Monaco, San Marino, and Vatican City were excluded due to a lack of data availability.

**Table 2:** Countries under evaluation for educational resource utilization efficiency.

Northern Europe	Eastern Europe	Western Europe	Southern Europe
Denmark	Belarus	Austria	Albania
Estonia	Bulgaria	Belgium	Andorra
Finland	Czechia	France	Croatia
Ireland	Hungary	Germany	Greece
Latvia	Poland	Luxembourg	Italy
Lithuania	Moldova	Netherlands	Portugal
Norway	Romania	Switzerland	Serbia
Sweden	Russian Federation		Slovenia
United Kingdom	Slovakia		Spain
	Ukraine		

## 5. Results and discussion

### 5.1 Super-SBM results

**Fig 1:**
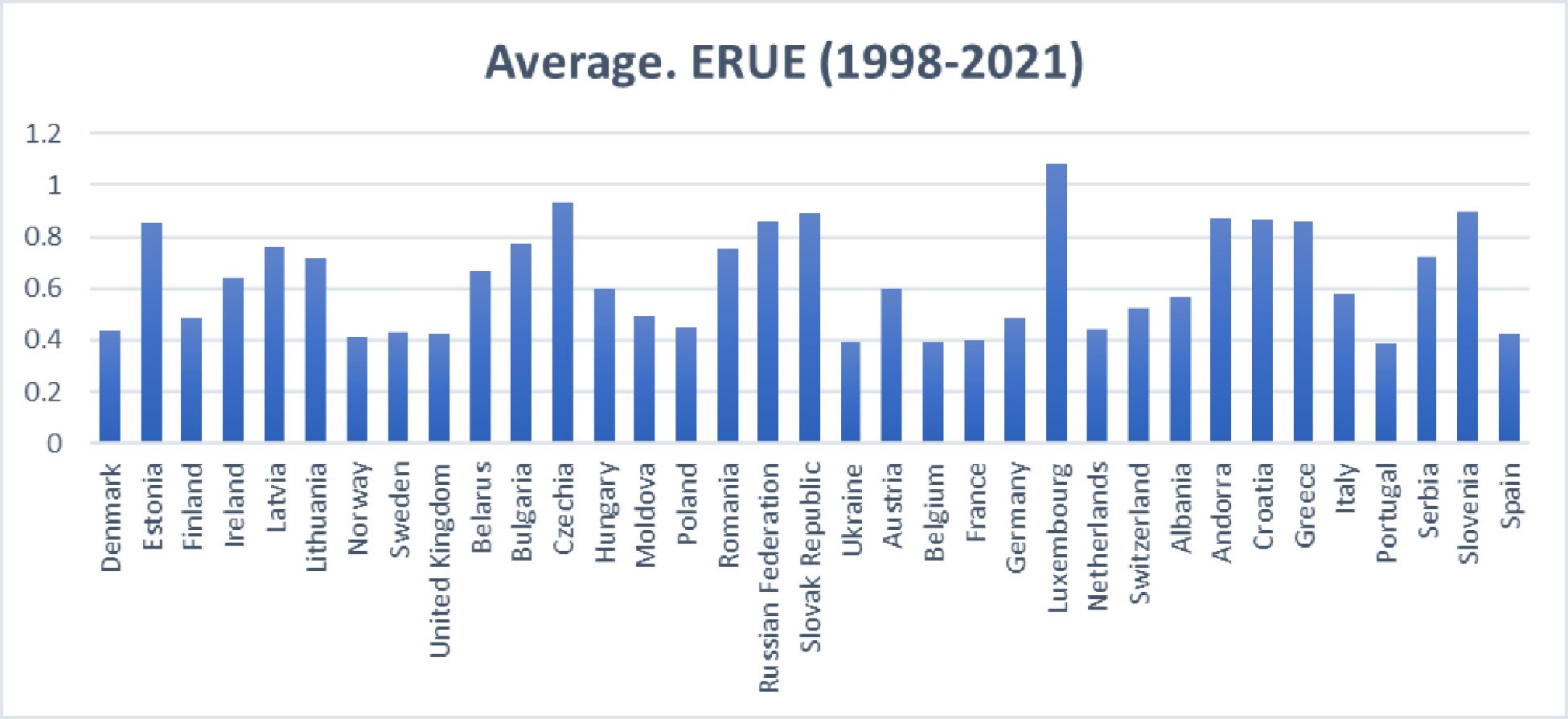
Average ERUE in European countries.

This study applied the super SBM model to evaluate the ERUE in 35 European countries Using the inputs and outputs discussed in Table 1. Results in Fig 1 show that the ERUE scores of Luxembourg, Czechia, Slovenia, Slovak Republic, Andorra, Croatia, Russian Federation, Greece, and Estonia range from 0.80 to 1.0813. It makes them a top performer in the 35 countries under evaluation. While Bulgaria, Latvia, Romania, Serbia, Lithuania, Belarus, Ireland, Hungary, Austria, Italy, Albania, and Switzerland scored between 0.50 and 0.80. Their performance is counted to be intermediate among the European countries under evaluation. Finally, Moldova, Germany, Finland, Poland, Netherlands, Denmark, Sweden, United Kingdom, Spain, Norway, France, Belgium, Ukraine and Portugal scored less than 0.50 and found to be least efficient in education resources utilization. Countries with lower efficiency in education can improve their utilization of resources by implementing various strategies. These strategies include making decisions based on data, optimizing the allocation of resources, investing in the training of teachers and innovation in the curriculum, reducing the number of students who drop out, involving parents and communities, monitoring performance, implementing targeted interventions, establishing partnerships between the public and private sectors, promoting inclusivity and equity, ensuring quality assurance, conducting educational research, engaging in long-term planning, improving financial efficiency, and fostering international collaboration. When implemented together, these measures result in improved educational outcomes and enhanced efficiency in the allocation of resources [71, 72].

### 5.2 Meta frontier analysis results

**Fig 2:**
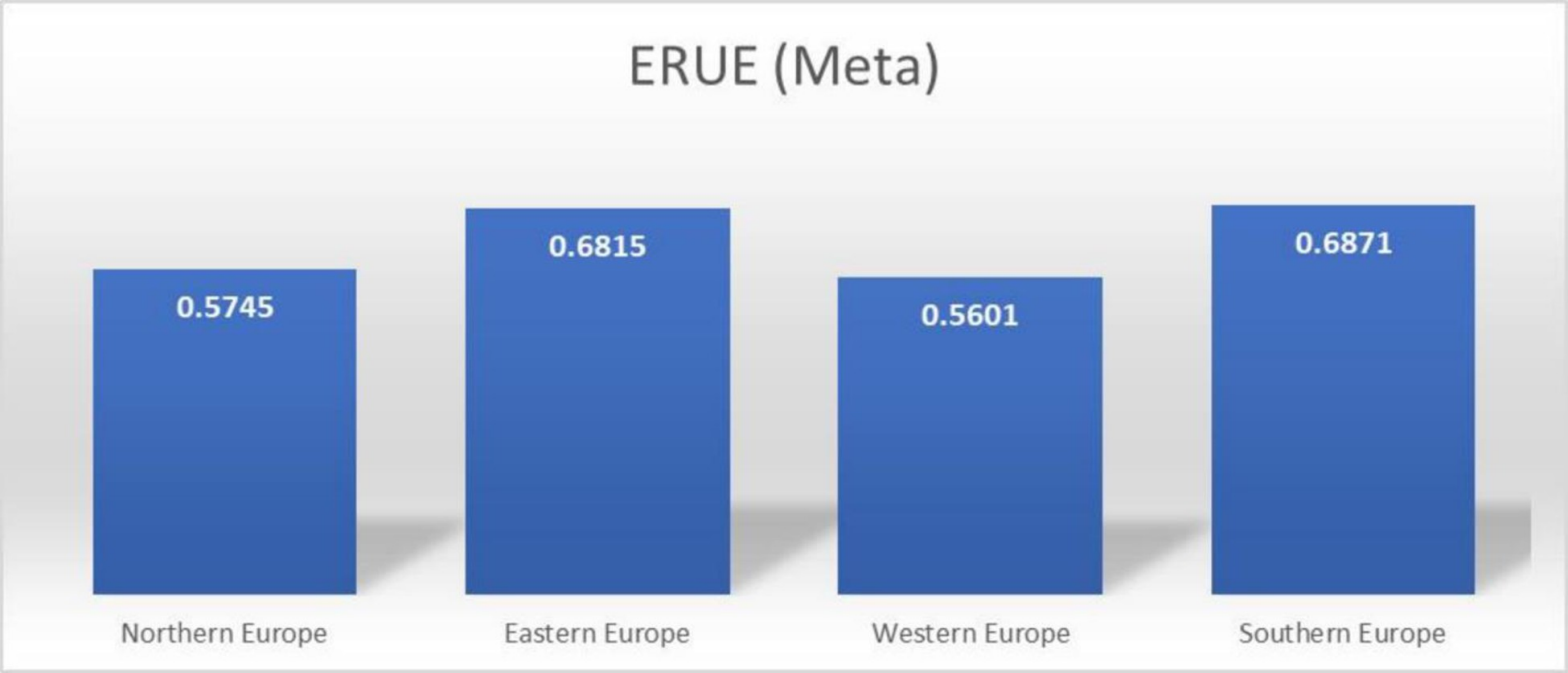
Average ERUE (Meta frontier) in four different regions of Europe.

To evaluate the efficiency of the use of educational resources across a variety of European countries, the Meta Frontier Analysis was conducted. Insightful conclusions can be drawn about the efficiency with which these countries allocate and use educational resources by employing this analytic approach, which incorporates both the meta and group frontier studies. The data in Table 3 showcases information about the Efficiency of Resource Utilization in Education (ERUE) across 35 European countries from 1998–2021. These countries are further classified into several regions, namely Northern Europe, Eastern Europe, Western Europe, and Southern Europe. Table 3 and Fig 2 present the mean scores of (ERUE) for each geographical region and the aggregate average for all countries. The scores measure the efficacy with which these nations employ their educational resources, with higher values denoting greater resource usage efficiency. The mean ERUE for 1998–2021 is 0.6312, indicating an inefficiency of 36.88% in educational resource utilization in European countries. Results revealed that ERUE in 2020 is at its highest level, with an average score of 0.73.

**Table 3:** Education resources utilization efficiency (Meta frontier) in 35 European countries.

Year	Northern Europe	Eastern Europe	Western Europe	Southern Europe	Avg All
1998	0.6130	0.7485	0.6659	0.7291	0.6921
1999	0.6299	0.7539	0.6591	0.7267	0.6961
2000	0.6369	0.8321	0.656	0.728	0.7199
2001	0.6198	0.6834	0.6369	0.7165	0.6662
2002	0.6162	0.6684	0.6352	0.735	0.6655
2003	0.5907	0.638	0.6201	0.7011	0.6385
2004	0.5559	0.5648	0.5745	0.6338	0.5822
2005	0.5982	0.6402	0.6066	0.6381	0.6221
2006	0.5962	0.6143	0.5786	0.6349	0.6078
2007	0.5137	0.5920	0.5515	0.5834	0.5615
2008	0.4896	0.5854	0.5385	0.5551	0.5436
2009	0.4150	0.6186	0.5072	0.518	0.5181
2010	0.6679	0.7438	0.5639	0.7696	0.695
2011	0.6048	0.7017	0.5525	0.7099	0.649
2012	0.463	0.7831	0.4842	0.7396	0.6298
2013	0.3447	0.6976	0.4379	0.4964	0.5032
2014	0.4632	0.6394	0.487	0.7485	0.5917
2015	0.5473	0.7959	0.5077	0.7124	0.6529
2016	0.5235	0.6976	0.4787	0.6615	0.5998
2017	0.5585	0.6364	0.4816	0.6099	0.5786
2018	0.6414	0.6178	0.4831	0.7846	0.6398
2019	0.6421	0.6478	0.491	0.7296	0.636
2020	0.7212	0.7318	0.6207	0.822	0.7300
2021	0.7357	0.7239	0.6245	0.8069	0.7284
**Avg.**	**0.5745**	**0.6815**	**0.5601**	**0.6871**	**0.6312**

Similarly, in 2013, it was at its lowest level, with an efficiency score of 0.5032. The available data facilitates a comparative examination of resource efficiency across various European regions and temporal periods. As an illustration, the data reveals that Southern Europe continuously exhibits superior average ERUE scores (0.6871) compared to other regions, indicating higher efficiency in utilizing educational resources. Conversely, Western Europe tends to have lower scores (0.5601) in this regard. The ERUE scores of Northern and Eastern Europe are 0.5745 and 0.6815, respectively.

**Fig 3:**
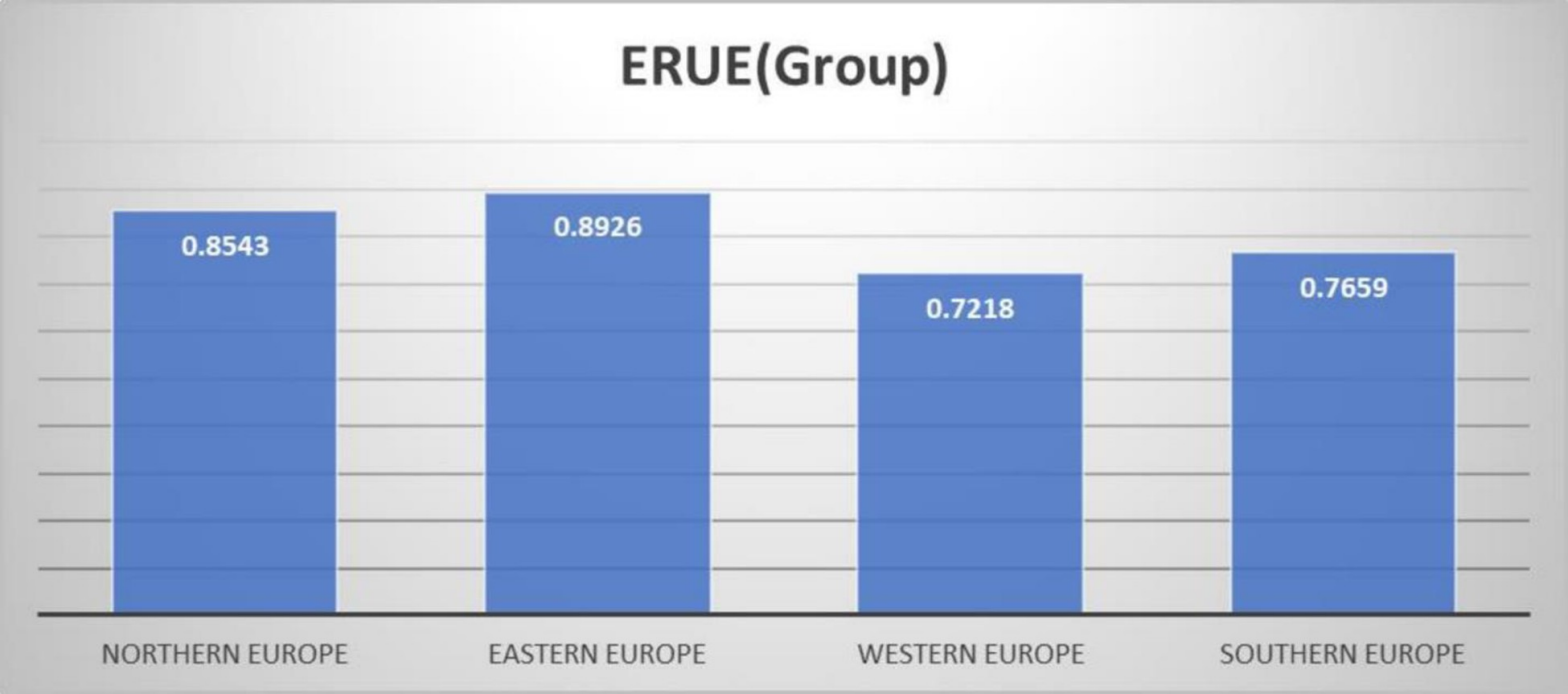
Average ERUE (group frontier) in four different regions of Europe.

Table 4 and Fig 3 explain European countries’ performance (ERUE) in their particular group. Table 4 provides a complete overview of the Efficiency of Resource Utilization in Education (ERUE) in 35 European nations from 1998 to 2021. The countries are categorized into discrete regions, namely Northern Europe, Eastern Europe, Western Europe, and Southern Europe, and their ERUE scores are systematically documented. The scores are indicators of the extent to which these nations effectively utilize their educational resources, with higher values indicating a better efficiency level. The column "Avg All" provides a comprehensive average ERUE score for all countries in each corresponding year, indicating an average ERUE of 0.816 for the study period. This observation indicates that, on average, European nations have a comparatively elevated degree of efficiency in allocating and utilizing their educational resources. Compelling patterns and trends are revealed after a more thorough data analysis. Eastern Europe has a leading position in ERUE ratings, consistently surpassing 0.9. It indicates a remarkable level of efficiency in resource consumption.

**Table 4:** ERUE (group frontier) in 35 European countries.

Year	Northern Europe	Eastern Europe	Western Europe	Southern Europe	Avg All
1998	1.021	0.9594	0.9831	0.7836	0.9348
1999	1.013	0.954	0.9758	0.7811	0.9291
2000	0.9624	0.9496	0.9287	0.7755	0.9039
2001	0.9149	0.9694	0.9189	0.7656	0.8929
2002	0.9295	0.9178	0.8982	0.7809	0.8817
2003	0.9302	0.961	0.8357	0.7661	0.8779
2004	0.9066	0.7942	0.8257	0.7464	0.8171
2005	0.9083	0.9685	0.8202	0.6947	0.853
2006	0.9134	0.7985	0.8198	0.701	0.8073
2007	0.9307	0.7796	0.7737	0.7138	0.8003
2008	0.8969	0.7679	0.7404	0.8003	0.8039
2009	0.8587	0.8359	0.5076	0.8437	0.7781
2010	0.7583	0.8897	0.8792	0.7677	0.8224
2011	0.8738	0.8453	0.8881	0.7564	0.8383
2012	0.6539	0.915	0.4858	0.9016	0.7586
2013	0.743	0.9214	0.4722	0.4601	0.6671
2014	0.6338	0.8798	0.487	0.8801	0.7381
2015	0.7569	0.9276	0.5829	0.7205	0.7615
2016	0.7656	0.8533	0.483	0.7729	0.736
2017	0.7573	0.8554	0.4844	0.7416	0.7267
2018	0.7595	0.8255	0.4879	0.8462	0.7463
2019	0.7609	0.9442	0.492	0.7535	0.7576
2020	0.9271	0.9493	0.7765	0.8347	0.8796
2021	0.927	0.9603	0.7765	0.7934	0.8721
**Avg.**	**0.8543**	**0.8926**	**0.7218**	**0.7659**	**0.816**

On the other hand, Southern Europe has initially lower scores but demonstrates a consistent improvement through time, indicating a positive trend in the efficiency of educational resources. Western Europe also exhibits a significant enhancement, as seen by the increase in ERUE scores, but it was still found to be the least efficient region in Europe, with a score of 0.7218. This upward trajectory indicates a favorable pattern in the region’s resource efficiency. Northern Europe often exhibits moderate ERUE scores and secures an average score of 0.8543. The aggregated results presented in Table 4 provide significant insights into managing educational resources across several European regions and countries. These findings demonstrate variances in efficiency levels among regions and highlight favorable trends in resource usage in several areas.

**Fig 4:**
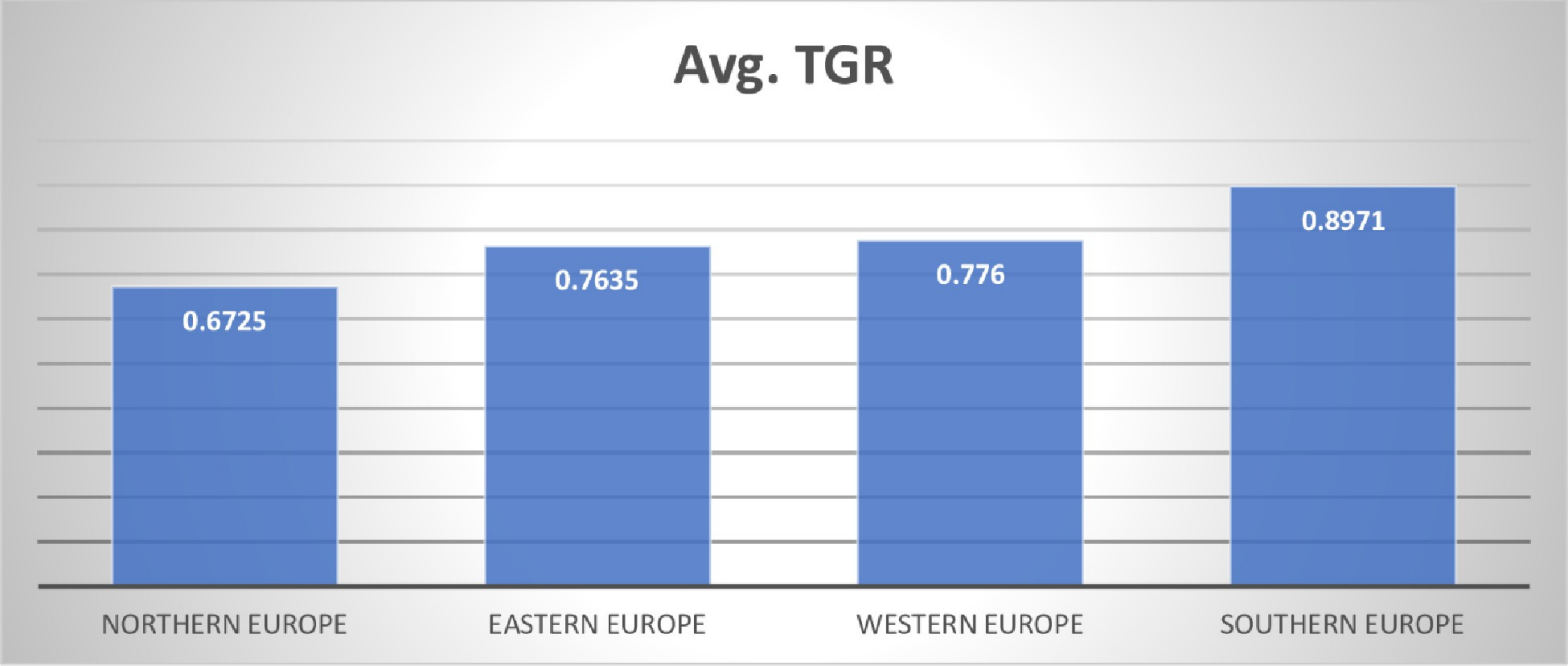
Average TGR in 4 regions of Europe.

Table 5 and Fig 4 present comprehensive data about the Technological Gap Ratio (TGR) in four distinct regions, namely Northern Europe, Eastern Europe, Western Europe, and Southern Europe, spanning the temporal range from 1998 to 2021. The technology difference Ratio (TGR) serves as a metric for assessing the disparity in technology advancements across different countries or regions. A greater TGR value signifies a less technological gap between the group and meta frontier. The "Avg. All" column displays the average (TGR) for all regions in each corresponding year. The TGR in 2017 is at its highest level, with an average score of 0.8817. The TGR value of Sothern Europe is 0.8971, the highest among all four regions. It illustrates that technological advancement in southern European is more advanced. Western Europe is ranked second with an average TGR value of 0.776.

**Table 5:** Technological Gap ratio (TGR) in four different regions (1998–2021).

Year	Northern Europe	Eastern Europe	Western Europe	Southern Europe	Avg. All
1998	0.6222	0.8122	0.6738	0.9361	0.7675
1999	0.6434	0.8229	0.6722	0.9355	0.7756
2000	0.687	0.8988	0.7077	0.9421	0.8172
2001	0.7121	0.7466	0.7004	0.9391	0.778
2002	0.6925	0.7734	0.7182	0.9472	0.7862
2003	0.6636	0.697	0.7859	0.9211	0.7638
2004	0.6418	0.8079	0.728	0.8518	0.7605
2005	0.6889	0.7085	0.7855	0.9196	0.7731
2006	0.6796	0.8159	0.7557	0.9105	0.7931
2007	0.5864	0.8049	0.783	0.8361	0.7523
2008	0.5775	0.8066	0.7917	0.7228	0.7232
2009	0.5111	0.7631	0.9991	0.655	0.7177
2010	0.8753	0.8688	0.6808	1.000	0.8709
2011	0.7217	0.8698	0.6547	0.9692	0.8142
2012	0.7469	0.8673	0.9964	0.8452	0.8565
2013	0.5205	0.7728	0.9136	1.0000	0.8098
2014	0.7611	0.7636	0.9999	0.8741	0.8386
2015	0.7405	0.8789	0.9246	1.0000	0.8849
2016	0.6967	0.8392	0.9907	0.8976	0.8479
2017	0.7456	0.7874	0.9938	0.8789	0.8415
2018	0.8369	0.7958	0.9895	0.9381	0.8817
2019	0.8354	0.7239	0.9977	0.9891	0.8755
2020	0.789	0.8104	0.8228	1.000	0.8573
2021	0.8032	0.7943	0.8303	1.000	0.8652
**Avg**	**0.6725**	**0.7635**	**0.776**	**0.8971**	**0.8105**

Similarly, eastern and northern Europe are ranked third and fourth with an average TGR of 0.7635 and 0.6725, respectively. Implementing a comprehensive and diverse strategy to mitigate the disparities in technological resources within educational contexts throughout different regions is advisable. First and foremost, it is imperative to prioritize information sharing and collaboration cultivation, thereby promoting the convergence of different regions and facilitating the exchange of exemplary practices and technological proficiency. This process can be facilitated by establishing partnerships and initiatives that enable technologically advanced countries to support those nations facing significant technological disparities. Additionally, allocating resources towards developing technological infrastructure is imperative, particularly in areas characterized by larger Technological Gap Ratios (TGR). It may entail enhancing internet connectivity, providing access to contemporary devices, and improving digital learning platforms. Capacity building is an essential component that involves the creation of educational programs and training efforts specifically designed for instructors and students. These programs provide individuals with the essential skills and knowledge required to utilize technology in the educational process efficiently. In addition, stimulating innovation and bridging technological disparities can be stimulated by promoting research and development in regions characterized by significant technology gaps. Collaboration among academics, industry, and government is advocated to facilitate advancements. The implementation of supportive policies is of utmost importance. These policies may encompass several measures, including providing incentives such as tax benefits for enterprises that allocate resources towards educational technology and facilitating grants for schools to get contemporary learning equipment. Promote the establishment of international collaborations to facilitate the exchange of experiences and resources between nations or organizations that have effectively addressed the disparities in educational technology. Finally, the ongoing review and assessment of a given situation are crucial to pinpoint specific areas that require development. Utilizing data-driven insights facilitates the enhancement of strategies and the efficient allocation of resources. Through the combined implementation of these initiatives and the cultivation of a culture that embraces technology adoption and innovation, European regions have the potential to cooperatively address and reduce disparities in educational resources related to technology. It confers advantages to specific geographical areas and fosters a more egalitarian and technologically sophisticated European educational environment.

This investigation’s specific results explain why some European countries are more efficient than others with educational resources and point out other interesting trends and patterns. Table 6 shows the results of Meta Frontier Analysis for different European countries and regions. It focuses on Educational Resource Utilization Efficiency (ERUE) at the meta and group levels and the average technological gap ratio (TGR). At first, the analysis shows that the average ERUE of 35 European countries from 1998 to 2021 is 0.6312 at the meta-level. It illustrates that the European countries still have an inefficiency of 36.88% in their educational resources’ utilization. A two-pronged strategy can be explored to improve the efficiency of educational resource utilization in European countries. The first aspect involves optimizing inputs, while the second focuses on enhancing output through more effective operational practices.

**Table 6:** Meta frontier, group frontier, and TGR in educational resources of European countries.

Region	Country	ERUE (Meta)	ERUE (Group)	Avg. TGR
**Northern Europe**	Denmark	0.4387	0.5479	0.8007
	Estonia	0.856	1.3434	0.6372
	Finland	0.488	0.8785	0.5555
	Ireland	0.6397	1.0247	0.6243
	Latvia	0.7616	0.965	0.7892
	Lithuania	0.7174	0.9017	0.7956
	Norway	0.41	0.5317	0.7711
	Sweden	0.4332	0.6962	0.6222
	United Kingdom	0.4261	0.7994	0.533
**Avg. Northern European countries.**		**0.5745**	**0.8543**	**0.6725**
**Eastern Europe**	Belarus	0.6683	0.8435	0.7923
	Bulgaria	0.7733	1.0439	0.7408
	Czechia	0.9356	0.9654	0.9691
	Hungary	0.6014	0.7683	0.7828
	Moldova	0.4932	1.4057	0.3509
	Poland	0.4479	0.4666	0.9599
	Romania	0.7535	0.9266	0.8132
	Russian Federation	0.8612	0.8664	0.994
	Slovak Republic	0.8888	1.2068	0.7365
	Ukraine	0.3919	0.433	0.9051
**Avg. Eastern European countries.**		**0.6815**	**0.8926**	**0.7635**
**Western Europe**	Austria	0.5983	0.8397	0.7125
	Belgium	0.3944	0.437	0.9025
	France	0.3964	0.4811	0.8239
	Germany	0.4884	0.8738	0.5589
	Luxembourg	1.0813	1.0813	1
	Netherlands	0.4405	0.5288	0.833
	Switzerland	0.5215	0.8109	0.6431
**Avg. Western European countries.**		**0.5601**	**0.7218**	**0.776**
**Southern Europe**	Albania	0.5703	0.7344	0.7766
	Andorra	0.8742	0.7444	1.1744
	Croatia	0.8667	1.0027	0.8644
	Greece	0.8605	0.9355	0.9198
	Italy	0.5772	0.6356	0.9081
	Portugal	0.3881	0.4266	0.9098
	Serbia	0.7228	0.8303	0.8705
	Slovenia	0.8984	1.1323	0.7934
	Spain	0.4258	0.4514	0.9433
**Avg. Southern European countries.**		0.6871	0.7659	0.8971
**Avg All**		**0.6312**	**0.816**	**0.7735**

Reducing inputs necessitates strategically deploying resources, including government expenditure and teacher numbers, to focus funding toward programs that provide the most benefit effectively. It may entail reallocating budgets following evidence-based policies and optimizing administrative processes to reduce inefficiencies. Concurrently, enhancing output efficiency necessitates a concentrated effort in delivering high-quality education. The achievement of this goal can be facilitated through the implementation of novel pedagogical approaches, the integration of contemporary technological tools, and the augmentation of teacher professional development to optimize student educational achievements. Furthermore, frequent assessments and feedback mechanisms can serve as valuable tools in identifying areas requiring improvement and facilitating timely adjustments. By effectively managing the allocation of resources and optimizing operational efficiency, European countries can strive to enhance the efficiency of educational resource utilization, thereby positively impacting their education systems and the future of their societies. Luxembourg (1.0813), Czechia (0.9356), Slovenia (0.8984), Slovak Republic (0.8888), and Andorra (0.8742) are found to be top performers among all 35 countries for the study period. It demonstrates that these countries optimally used the educational resources to produce more output and remained the benchmark for other countries under evaluation. The ERUE has better results at the group level, with an average score of 0.816. The average technological gap between the group frontier and meta frontier is 0.7735.

Further elaborating on the different regions in Europe, we found that the average ERUE (0.6871) of Southern European countries is the highest among all four regions. While the ARUE of Eastern European countries is 0.6815, Northern European countries are 0.5745, and Western European countries are 0.5601. It illustrates that Southern European countries are top performers and utilize their resources optimally to achieve higher resource utilization and education efficiency. Based on the provided ERUE values, it can be deduced that Southern European countries exhibit higher efficiency in utilizing their educational resources. The region in question has demonstrated superior efficiency in allocating resources and delivering education, surpassing that of other regions. The observed phenomenon can be ascribed to the implementation of efficient resource allocation strategies, the formulation of successful educational policies, or other contributing elements that facilitate the optimization of education within these nations.

Group frontier results explain each country’s efficiency evaluation in its group. The average ERUE of Eastern European countries (0.8926) is comparatively higher than other European regions. The average ERUE of Northern European countries is 0.8543, Southern European countries are 0.7659, and Western European countries are 0.7218. Eastern European nations have superior average efficiency in this domain, while Western European nations demonstrate comparatively lower efficiency levels, as indicated by their respective average ERUE measurements. This information holds significant value for policymakers and other authorities interested in enhancing resource allocation and efficiency within the education sector or other relevant sectors. It is recommended that regions adopt a comprehensive and diversified approach to optimize the effectiveness of educational resource usage; it involves employing a data-driven approach to decision-making to identify areas that require enhancement, optimizing the allocation of resources with an emphasis on effective programs, and investing in teacher quality and professional development. Studies have proved that utilizing technology to deliver content and mitigate bureaucratic obstacles can optimize operational processes and lead to cost reduction [73, 74].

Furthermore, the implementation of inclusive education policies, the establishment of public-private partnerships, and the adoption of innovative teaching methods have the potential to enhance learning outcomes and optimize resource use. Long-term planning necessitates the inclusion of crucial elements such as infrastructure development, policy reforms, and public engagement to guarantee sustainable progress. International collaboration and the assimilation of global best practices have the potential to enhance and enhance local initiatives through the acquisition of knowledge and insights. By adopting these ideas, regions can strive towards a more effective and fair education system that empowers learners and optimizes existing resources [75, 76].

### 5.3 Malmquist productivity index results

The findings acquired from the examination are comprehensively presented in the results part of the Malmquist productivity index analysis for education resource utilization efficiency in European countries. This section provides a comprehensive analysis of the fluctuations in efficiency and productivity over time, offering insights into the dynamic nature of educational resource utilization within European countries. The analysis elucidates the principal patterns, accentuates noteworthy advancements or setbacks, and provides valuable perspectives on the underlying determinants influencing shifts in productivity. Table 7 displays the Malmquist Index (MI), Efficiency Change (EC), and Technology Change (TC) used to assess the utilization of educational resources in 35 European countries. These countries are further classified into four regions: Northern Europe, Eastern Europe, Western Europe, and Southern Europe. The Malmquist Index provides a means to analyze the overall dynamics of production and efficiency over a given period, where values greater than 1 signify positive advancements. Efficiency Change pertains to the evaluation of differences in the utilization of resources, specifically in terms of efficiency. On the other hand, Technology Change involves the assessment of improvements in technology. The countries under consideration have demonstrated a collective improvement in their productivity and efficiency in allocating educational resources over the period, as indicated by an average Malmquist Index (MI) of 1.0349. It illustrates that in the study period, European countries got 3.49% growth in educational resources utilization. The EC, with an average value of 1.0165, indicates a favorable pattern in the utilization of resources, demonstrating the collective endeavors to enhance educational processes. The dynamic efficiency witnessed a 1.65% growth over the study period. Furthermore, the average Technology Change (TC) value of 1.0181 highlights a persistent adoption of technical breakthroughs in education, indicating that European nations are incorporating sophisticated technologies into their educational frameworks. Technological change gets 1.81 percent growth. The TC (1.0181) > EC 1.0165 indicates that technology is the main determinant in productivity growth. The Northern European countries under consideration have demonstrated a collective improvement in their productivity and efficiency in allocating educational resources, as indicated by an average Malmquist Index (MI) value of 1.0389 for the examined period. Furthermore, the estimated average Efficiency Change (EC) of 1.0168 demonstrates a notable increase of 1.68% in education resource efficiency, which signifies a collective endeavor to enhance educational processes. The mean Technology Change (TC) score of 1.0217 is noteworthy, indicating a deliberate incorporation of state-of-the-art technology into the educational systems of these countries. It highlights their dedication to fostering innovation and leveraging technology in education, ultimately improving the efficiency of educational resource usage. Technology change is the main determinant in productivity growth in educational resources of Northern European countries. Table 7 further presents data on Eastern European countries, indicating significant advancements in the application of educational resources. The Eastern European countries analyzed in this study have collectively demonstrated an average Malmquist Index (MI) of 1.0248, indicating improved productivity and efficiency in allocating educational resources over the assessed period. In addition, the average Efficiency Change (EC) of 1.0147 indicates a noteworthy increase of 1.47% in resource efficiency. It demonstrates the collective endeavors made to enhance educational processes in this region. The Efficiency Change exhibits favorable patterns in the utilization of resources, while the average Technology Change (TC) value of 1.0100 highlights a dedication to incorporating technological improvements into educational institutions. The region’s steadfast commitment to innovation highlights its focus on improving efficiency and advancing technology in utilizing educational resources, ultimately improving education quality. Efficiency change in Eastern European countries is the main determinant of productivity growth. The Western European countries examined in this study demonstrate an average Malmquist Index (MI) of 1.0196, indicating an overall enhancement in productivity and efficiency in allocating educational resources during the assessed period. Furthermore, the calculated average Efficiency Change (EC) of 1.0113 demonstrates a significant increase of 1.13% in resource efficiency, highlighting the collective endeavors to enhance educational processes. The Efficiency Change exhibits a favorable trend in the usage of resources. At the same time, the average Technology Change (TC) value of 1.0082 underscores their dedication to incorporating technology improvements into their educational systems. It demonstrates a commitment to fostering innovation and utilizing technology in education, leading to improved efficiency and effectiveness in utilizing educational resources in Western European countries. Finally, the results on educational resource usage in Southern European countries indicate a significant advancement in this region. The nations collectively demonstrate a noteworthy enhancement in productivity and efficiency in allocating educational resources over the analyzed period, as evidenced by an average Malmquist Index (MI) of 1.0542. Moreover, the mean Efficiency Change (EC) of 1.0222 indicates a significant increase of 2.22% in resource efficiency, highlighting the collective endeavors to enhance educational processes in the given area. One notable aspect is the mean Technology Change (TC) score of 1.0313, which highlights their dedication to incorporating technological progressions within their educational frameworks. This statement highlights the commitment of individuals to fostering innovation and utilizing technology in education, resulting in improved efficiency and effectiveness in the allocation of educational resources in Southern European countries. Results conclude that Northern and Southern European countries kept superior technology, which helped them in productivity growth. While Eastern and Western European countries have higher efficiency changes in educational resource utilization. It illustrates that eastern and western European can benchmark the northern and southern European countries for technological advancement. They could acquire the technology from these regions to further enhance their educational resources’ productivity growth. On the other hand, southern and northern European countries can acquire the operational strategies of the educational sector to enhance their efficiency in educational resource utilization. Numerous research studies endorsed that technological development is a key factor in education resources utilization efficiency across the globe [77, 78].

**Table 7:** Malmquist Index, efficiency change and technology change in educational resources utilization of 35 European countries.

Region	Countries	MI	EC	TC
Northern Europe	Denmark	1.0206	1.0063	1.0142
	Estonia	1.0448	1.0227	1.0216
	Finland	1.0273	1.0189	1.0082
	Ireland	1.0574	1.0245	1.0321
	Latvia	1.0654	1.0019	1.0634
	Lithuania	1.0786	1.0474	1.0298
	Norway	1.0432	1.0322	1.0107
	Sweden	1.012	0.9975	1.0145
	United Kingdom	0.9999	0.9994	1.0005
**Avg. Northern European countries.**		1.0389	1.0168	1.0217
Eastern Europe	Belarus	1.0462	1.0232	1.0225
	Bulgaria	1.0679	1.0683	0.9996
	Czechia	0.9912	0.9815	1.0099
	Hungary	1.0721	1.048	1.023
	Moldova	1.0161	0.9921	1.0242
	Poland	1.0168	1.0113	1.0054
	Romania	0.9978	1.0425	0.9571
	Russian Federation	1.0423	0.9811	1.0624
	Slovak Republic	0.9967	1.0081	0.9887
	Ukraine	0.9984	0.9912	1.0073
**Avg. Eastern European countries.**		1.0248	1.0147	1.0100
Western Europe	Austria	1.0612	1.0426	1.0178
	Belgium	1.0103	1.0056	1.0047
	France	1.0136	1.0108	1.0028
	Germany	1.0316	1.0235	1.0079
	Luxembourg	1.003	1.0003	1.0027
	Netherlands	1.0083	1.0005	1.0078
	Switzerland	1.0094	0.996	1.0135
**Avg. Western European countries.**		1.0196	1.0113	1.0082
Southern Europe	Albania	1.0335	1.0235	1.0098
	Andorra	1.0008	1.0132	0.9878
	Croatia	1.0153	1.01	1.0052
	Greece	1.0565	0.996	1.0607
	Italy	1.1857	1.07	1.1081
	Portugal	1.0375	1.021	1.0162
	Serbia	1.0746	1.017	1.0566
	Slovenia	1.0647	1.0316	1.0321
	Spain	1.0227	1.0174	1.0052
**Avg. Southern European countries.**		1.0542	1.0222	1.0313
**Avg all**		**1.0349**	**1.0165**	**1.0181**

Moreover, Italy (1.1857), Lithuania (1.0786), and Serbia (1.0746) were found to have higher average MI scores over the study period (1998–2021). It illustrates that these countries are most dynamic and productive in using educational resources. Italy, Bulgaria, and Hungary have the highest EC in all 35 European countries. Italy, Latvia, and the Russian Federation secured the superior TC over the study period. Enhancing the Malmquist Productivity Index (MI), Technology Change (TC), and Efficiency Change (EC) in the allocation of educational resources in inefficient European countries requires the implementation of an extensive plan. It involves enhancing the educational infrastructure, encompassing physical facilities and technical resources, to create an optimal learning environment for students. Equally significant is the allocation of resources towards teacher training and professional development, as this enables educators to acquire the necessary competencies to deliver high-quality education effectively. To advance educational practices, adopting technological integration and revising curricula to cultivate critical thinking and problem-solving abilities is imperative. Enhancing educational results can be achieved by implementing robust assessment systems, equitable allocation of resources, and fostering cooperation with public and private partners. Incorporating inclusivity, early childhood education, and international cooperation constitutes essential components of this comprehensive approach. It is imperative to prioritize long-term planning, implement legislative reforms, and demonstrate a steadfast dedication to continual development to enhance educational outcomes in inefficient European countries [79, 80].

### 5.4 Kruskal Wallis test results

**Fig 5:**
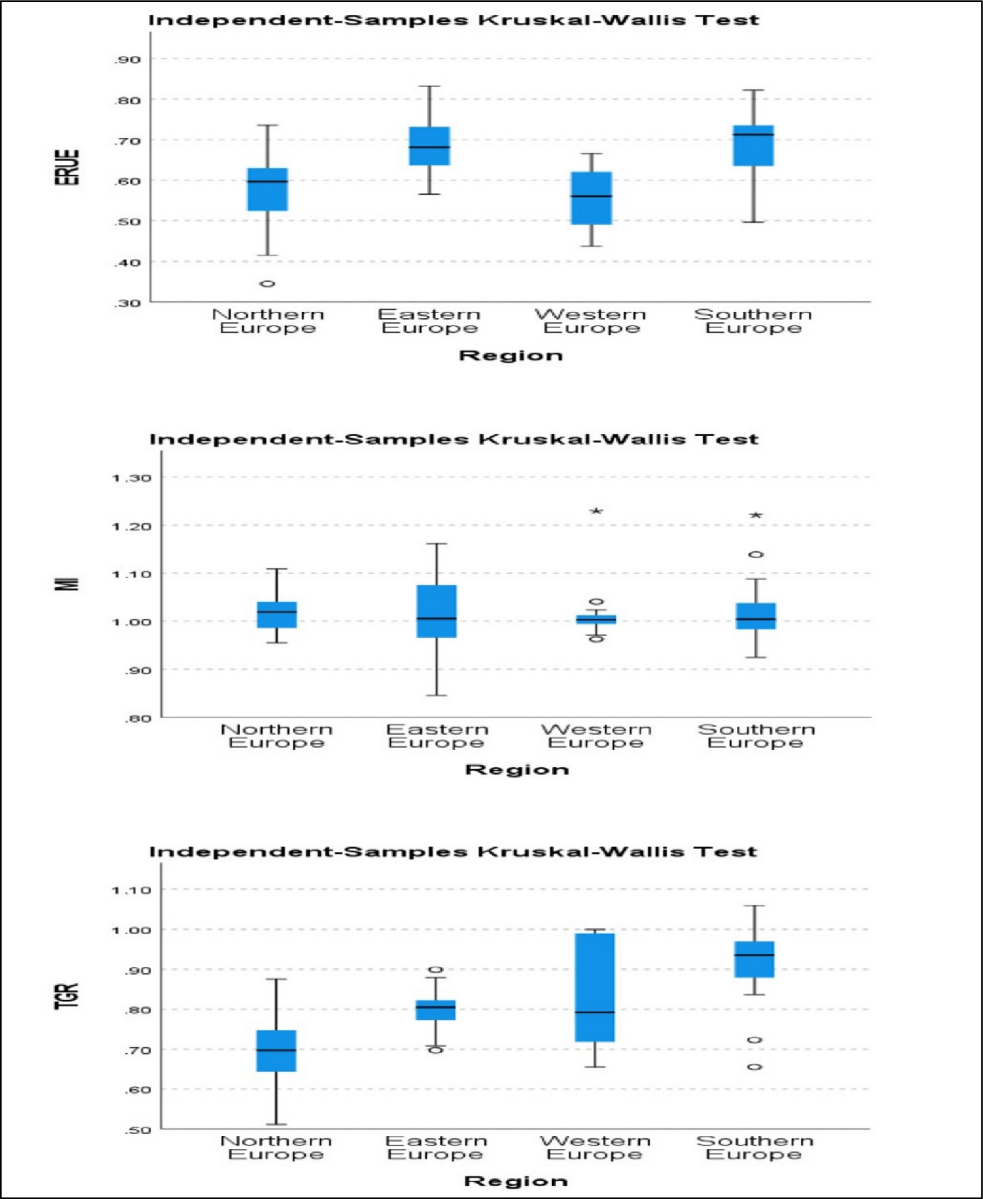
ERUE, MI and TGR distribution in 4 European regions.

The findings in Sections 5.1, 5.2, and 5.3 illustrate the heterogeneity of ERUE, MI, and TGR across four distinct European regions. However, the critical question about the statistical significance of these differences is paramount in ensuring the reliability of these results. To address this concern, the present study employed the Kruskal-Wallis test to assess whether statistically significant disparities exist among the four European regions concerning ERUE, MI, and TGR. Table 8 and Fig 5 display the results of the Kruskal-Wallis Test. The significance value associated with the first null hypothesis is 0.001, less than the conventional threshold of 0.050.

**Table 8:** Kruskal–Wallis test results.

**Hypothesis Test Summary**				
	**Null Hypothesis Test Sig. Decision**			
1	The average ERUE scores are the same across four different European regions	Independent-Samples Kruskal–Wallis Test	.001	Reject the null hypothesis.
2	The average MI scores are the same across four different European regions.		.711	Retain the null hypothesis
3	The average TGR scores are the same across four different European regions.		.001	Reject the null hypothesis

Asymptotic significances are displayed. The significance level is .050.

Consequently, we reject the first null hypothesis, which posits that the mean ERUE scores are uniform across the four European regions. These outcomes substantiate the contention that the efficiency of educational resources varies significantly among these regions. Conversely, the significance value for the second hypothesis is 0.711, surpassing the 0.050 threshold. Consequently, we retain the second hypothesis, which postulates that the mean MI scores do not differ significantly across the four European regions. It implies that there is no substantial variance in the growth of educational resource productivity across these regions, as they all exhibit comparable levels of growth in educational resource utilization over the study period. The significance value associated with the third and final hypothesis is 0.001. Thus, we reject the third hypothesis, which suggests that the mean TGR scores are uniform across the four European regions. This finding indicates that the utilization of technology in the educational sector varies significantly across these regions, underlining the heterogeneity in technological adoption in European educational contexts.

## 6. Conclusion and policy recommendations

Efficiently utilizing educational resources is crucial because it affects the quality of education and has broader ramifications for society and the economy. It ensures that students receive the best education possible, enhancing learning outcomes and a more skilled labor force. In addition, resource efficiency promotes social justice by making quality education accessible to all, regardless of socio-economic status, thereby nurturing inclusivity and reducing educational disparities. It also promotes educational innovation, propelling progress and preparing individuals for a constantly changing world. Education systems that effectively contribute to a country’s global competitiveness, economic growth, and environmental sustainability. European countries are undertaking significant endeavors to enhance the efficiency of educational resource utilization. These programs encompass the utilization of data-driven decision-making, the provision of teacher training, the promotion of curriculum innovation, the reduction of dropout rates, and the integration of digital technology. Public-private partnerships, the promotion of diversity, and the provision of open-access materials are further tactics implemented to improve the quality of education and the allocation of resources. Differences in efficiency, productivity growth, and technology heterogeneity among European countries are notable obstacles to the equitable utilization of educational resources. The collective endeavors mentioned enhance educational achievements, foster economic expansion, and bolster Europe’s position in the global market. However, the success in this mission of ERUE, Productivity growth, and technological heterogeneity among different European regions are unexplored and need comprehensive investigation.

This study employed DEA Super-SBM, Meta-frontier Analysis, and Malmquist productivity index for 1998–2021 to evaluate the educational resources utilization efficiency, production technology heterogeneity, and resource productivity growth in 35 European countries. Results illustrate that the mean ERUE for 1998–2021 is 0.6312, indicating an inefficiency of 36.88% in educational resource utilization in European countries. Southern Europe continuously exhibits superior average ERUE scores (0.6871) compared to other regions, indicating a higher efficiency level in the utilization of educational resources. Conversely, Western Europe tends to have lower scores (0.5601) in this regard. The ERUE scores of Northern and Eastern Europe are 0.5745 and 0.6815, respectively. Luxembourg (1.0813), Czechia (0.9356), and Slovenia (0.8984) are found to be the top three performers in terms of ERUE level. The results of the group frontier demonstrate that Eastern Europe is superior among all, while Northern Europe, Southern Europe, and Western Europe are ranked second, third, and fourth, respectively. The technology gap ratio value is highest in Southern Europe. It demonstrates that southern European countries used the most advanced technology in education resource utilization. While Western, Eastern, and Northern Europe secure 2^nd^, 3^rd,^ and 4^th^ positions, respectively, to demonstrate their technological level.

The analysis of European countries’ educational resource allocation and productivity reveals a collective improvement, indicated by an average Malmquist Index (MI) of 1.0349. Over the study period, there was a 3.49% growth in educational resource utilization, underlining the region’s commitment to enhancing efficiency. Technology adoption played a pivotal role, with a 1.81% growth in the Technology Change (TC) index, making it the main driver of productivity growth. Northern European countries led in productivity gains with an MI of 1.0389, emphasizing efficiency and technology adoption. Eastern European nations followed suit, with an MI of 1.0248, highlighting efficiency improvements and technology integration. Western European countries also improved, with an MI of 1.0196, focusing on efficiency and technology enhancements. Notably, Southern European countries showed significant advancement, boasting an MI of 1.0542, driven by efficiency gains and a strong commitment to technology adoption. These findings suggest opportunities for knowledge exchange between regions, with Northern and Southern Europe excelling in technology and Eastern and Western Europe demonstrating resource efficiency expertise. Italy (1.1857), Lithuania (1.0786), and Serbia (1.0746) are found to have higher average MI scores over the study period (1998–2021). It illustrates that these countries are most dynamic and productive in using educational resources. Finally, the Kruskal Wallis test proved that ERUE and TGR in 4 different regions of Europe are heterogeneous. In contrast, the MI in European regions isn’t found to be significantly different. Effectively managing educational resources poses a significant objective and a multifaceted dilemma for European nations. The study’s policy recommendations have substantial consequences for policymakers and stakeholders in the field of education.

One of the primary suggestions is the crucial need to prioritize providing education of superior quality. Efforts should be focused on channeling efficiency benefits towards strengthening the overall quality of education, aiming to improve learning outcomes and cultivating a competitive and adaptive workforce. It involves allocating resources towards highly skilled educators, updated curricula, and advanced educational tools. By cultivating a milieu that prioritizes high standards and quality in the realm of Education, European countries may effectively equip their population with the necessary skills and knowledge to effectively navigate the complexities and prospects of the contemporary day.

Simultaneously, adopting a fair and just method of distributing resources is imperative to foster social justice and inclusiveness. Education is a potent instrument for mitigating socio-economic inequalities, and via equitable resource allocation, European nations may provide inclusive possibilities for individuals irrespective of their socio-economic backgrounds. The dedication to inclusivity not only yields advantages for marginalized people but also enhances the cohesion of societies. Inefficient countries and regions in Europe can adopt the strategies and resource allocation methods from efficient European countries and regions.

Another crucial recommendation is to promote educational innovation. In an era characterized by swift transformations, educational systems must constantly adapt to maintain relevance. It is imperative for policymakers to actively promote the adoption of innovative pedagogical approaches and the incorporation of digital technologies inside educational institutions. The integration of digital technology in education enriches students’ educational journey and empowers them with the essential digital literacy competencies required to thrive in contemporary knowledge-driven economies. The study found that the production technologies of southern Europe are more advanced and current. Therefore, the other regions can acquire the production technologies to improve their technological level in the education sector.

Furthermore, given the significant impact of education on a nation’s competitiveness, economic growth, and environmental sustainability, European countries must persist in allocating resources toward initiatives that enhance educational efficacy. By strategically optimizing resource allocation, nations can effectively position themselves in the global economy while simultaneously reducing the environmental impact of educational institutions.

Utilizing data-driven decision-making is a highly valuable approach to optimize the allocation of resources. It is imperative for policymakers to actively advocate for the use of data in shaping educational policies, enabling educational institutions to effectively identify and address areas in need of development while making decisions grounded in empirical facts. The utilization of a data-driven strategy not only serves to optimize efficiency but also facilitates the ability of educational institutions to respond effectively to dynamic situations. Enhancing educational quality necessitates allocating resources toward teacher training and curricular innovation. Highly skilled educators play a pivotal role in the foundation of any education system, and by providing them with contemporary teaching approaches and methodologies, nations can enhance the overall standard of education. Furthermore, updating curricula to align with current challenges and possibilities is essential to provide students with an education that is both pertinent and captivating.

Addressing the issue of high dropout rates is an additional opportunity to improve overall efficiency. It is imperative to ensure that students who continue their education receive optimal resources and that efforts to reduce dropout rates are prioritized to maximize the effectiveness of educational spending. Public-private partnerships (PPPs) can facilitate the development of creative solutions and enhance the availability of resources. It is imperative for policymakers to proactively facilitate partnerships between public and private institutions to infuse the educational sector with novel perspectives and resources. Ultimately, facilitating the interchange of knowledge throughout different locations is vital for collective advancement. Through exchanging best practices and disseminating lessons learned, European nations can derive reciprocal advantages from one another’s achievements and difficulties. Northern and Southern European countries, renowned for their advanced technological capabilities, have the potential to contribute their experience in technology adoption. Conversely, Eastern and Western European countries, recognized for their proficiency in resource efficiency, stand to gain significant insights in this domain.

Although this research offers significant contributions to understanding the efficiency, productivity growth, and technology heterogeneity related to the usage of educational resources in European countries between 1998 and 2021, it is crucial to recognize several constraints inherent in our study. The study’s chronological limitations may hinder its capacity to encompass the most recent advancements in swiftly moving educational systems. Furthermore, the dependence on data, which can exhibit disparities in both quality and accessibility across different countries, increases the possibility of introducing bias. From a methodological standpoint, it is important to acknowledge that the techniques we have selected, namely DEA, Super-SBM, Meta-frontier Analysis, and Malmquist productivity index, provide valuable insights. However, it is crucial to recognize that these techniques are accompanied by certain assumptions and restrictions. The categorization of European countries into regions conducted in this study may potentially oversimplify the intricate variations that exist within these regions. Consequently, it is necessary to conduct further examination into the dynamics at the sub-regional level. In order to overcome these constraints, future investigations may consider doing more detailed regional analyses, employing longitudinal study designs, integrating qualitative research methods, evaluating the effects of enacted policies, and examining comparative viewpoints with nations outside of Europe. Furthermore, the inclusion of sustainability in the management of educational resources would serve to augment the practical significance of the study. These aforementioned factors will enhance the depth of comprehension regarding the exploitation of educational resources and provide valuable guidance for formulating more efficacious policy recommendations for European countries.
